# Nonlinear gravity electro-capillary waves in two-fluid systems: solitary and periodic waves and their stability

**DOI:** 10.1007/s10665-021-10182-8

**Published:** 2022-03-09

**Authors:** H. Broadley, D. T. Papageorgiou

**Affiliations:** 1 School of Mathematics, University of Manchester, Manchester, M13 9PL UK; 2 Department of Mathematics, Imperial College London, 180 Queen’s Gate, London, SW7 2BZ UK

**Keywords:** Electro-capillary waves, Nonlinear waves, Solitary waves, Two-fluid flows

## Abstract

Starting from the Euler equations governing the flow of two immiscible incompressible fluids in a horizontal channel, allowing gravity and surface tension, and imposing an electric field across the channel, a nonlinear long-wave analysis is used to derive a $2\times 2$ system of evolution equations describing the interface position and a modified tangential velocity jump across it. Travelling waves of permanent form are shown to exist and are constructed in the periodic case producing wave trains and the infinite case yielding novel gravity electro-capillary solitary waves. Various regimes are analysed including a hydrodynamically passive but electrically active upper layer, pairs of perfect dielectric fluids and a perfectly conducting lower fluid. In all cases, the presence of the field produces both depression and elevation waves travelling at the same speed, for given sets of parameters. The stability of the non-uniform travelling waves is investigated by numerically solving appropriate linearised eigenvalue problems. It is found that depression waves are neutrally stable whereas elevation ones are unstable unless the surface tension is large. Stability or instability is shown to be linked mathematically to the type of local eigenvalues of the nonlinear flux matrix used to obtain travelling and solitary waves; if these are real (hyperbolic flux matrix), the system is stable, and if they are complex (elliptic), the system is unstable. The latter is a manifestation of Kelvin–Helmholtz instability in electrified flows.

## Introduction

Two-fluid immiscible flows can be found in numerous applications and at different scales. On the micro-scale, inertia is absent, and in addition to viscously dominated dynamics, other physical mechanisms such as surface tension become dominant. On the other hand at large scales (e.g. geophysical applications), viscosity is absent and so is surface tension, with large-scale motions underpinned by gravity-driven waves as well as density variations. The present work aims to study scales between these two, namely laboratory experiments that have considerable inertia and for which surface tension and other external fields, such as electric fields are present.

An idealised mathematical model considers two inviscid, immiscible fluids in a channel and seeks to describe the nonlinear waves that can emerge. In the presence of gravity, asymptotic analysis was used to produce the Miyata–Choi–Camassa model—see Miyata [1] and Choi and Camassa [2]—a system of reduced-dimension evolution equations. More recent advances include the presence of three layers and the construction of periodic and solitary waves, see Lopes-Barros et al. [3] and references therein. It is quite likely that all such waves are susceptible to Kelvin–Helmholtz instabilities, but water tank experiments by Carr et al. [4] show clearly that the coherent structures survive and are the dominant essential feature of the flow.

The present study extends the systems described above in two ways: first by including surface tension as a dispersive regularisation of short-wave Kelvin–Helmholtz instabilities and second, an electric field is imposed across the channel with the channel walls acting as electrodes that support a voltage potential across it. A seminal reference in the field of interfacial electrohydrodynamics is Melcher [5], where theory and experiments are used to describe the effects of electric fields on fluid–fluid interfaces (see also Melcher and Taylor [6], Saville [7]). For a recent review on nonlinear waves found in electrohydrodynamic multfluid flows, see Papageorgiou [8]. Linear theory predicts that generally an electric field acting normal to the undisturbed interface provides instability, whereas tangential ones stabilise the flow (dispersively in the case of ideal fluids). In the latter scenario, recent work by Zhan and Yang [9] proves local well-posedness of the Kelvin–Helmholtz problem, and in fact for two-dimensional flows, the presence of a tangential electric field is sufficient even in the absence of surface tension.

Here, we are concerned with vertical electric fields in channel flows as originally formulated by Melcher [5], and we mention some relevant work. Long-wave models were derived and studied for single-layer flows [10], and it was shown that the electrically induced instabilities can produce singular behaviour such as blow-up. Weakly nonlinear Stokes wave analyses, including construction of Wilton ripple travelling waves, as well as fully nonlinear computations were carried out for single-layer electrified problems in [11], and Gleeson et al. [12] show how a fifth-order Kortweg–de Vries Benjamin–Ono equation derives from such electrified problems. Wang [13] also developed a fully nonlinear model from which the governing equations for two-dimensional gravity–capillary waves under a normal electric field were derived. Gao et al. [14] consider a perfect dielectric fluid of infinite depth bounded above by a perfectly conducting gas, and construct fully nonlinear gravity–capillary waves and study their stability—mathematically one region needs to be accounted for and conformal mapping techniques are appropriate. Doak et al. [15] studied both linear and weakly nonlinear theories of interfacial gravity–capillary waves on the surface between two dieletric fluids, with the upper fluid being hydrodynamically passive. When multiple regions are present, the problems are more complicated, and in this work, we begin a study of such systems where the effects of gravity, surface tension, electric fields and tangential velocity jumps are accounted for. We note that in the absence of an electric field analogous simpler systems emerge and were studied in [16]; solitary waves were constructed including some exact solutions for a discrete set of parameters. In the present study, we consider the stability of such solitary waves and our results indicate that they are linearly stable.

The rest of the paper is organised as follows. Section 2 states the governing equations and boundary conditions in their general form. Section 3 carries out a long-waves analysis to produce reduced-dimension model equations that allow the interface to scale with the channel height. Section 4 constructs two-fluid solitary waves for a range of physical situations, and Sect. 5 constructs finite-period travelling waves. The stability of these non-uniform coherent structures is considered in Sects. 6 and 7 we provide a discussion.

## Governing equations

**Fig. 1:**
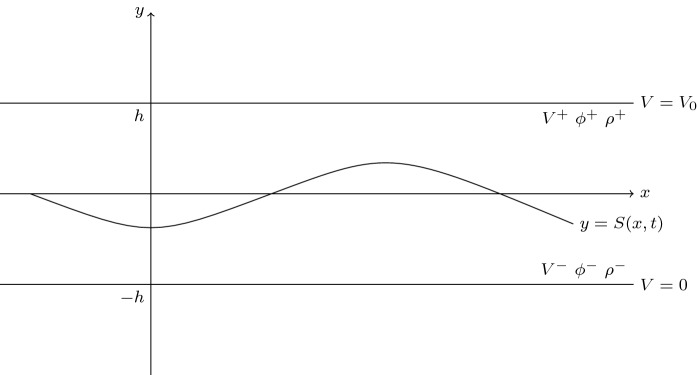
A schematic of the incompressible, inviscid two-fluid system

Consider two immiscible irrotational, incompressible, inviscid fluids in a channel of height 2*h* and infinite horizontal extent. Using a Cartesian coordinate system (*x*, *y*), the smooth and parallel channel walls are located at $y=\pm h$, and the undisturbed interface is at $y=0$, i.e. the fluids have equal mean thickness *h*. Two-dimensional flows are also assumed. A schematic is given in Fig. 1 which shows the general situation with a deformed interface given by $y=S(x,t)$. Taking the fluids to be perfect dielectrics, we introduce an electric potential *V*(*x*, *y*, *t*) within the channel by imposing constant voltages $V(x,-h)=0$ and $V(x,h)=V_0$ at the lower and upper wall electrodes, respectively. In the absence of an electric field, the problem was studied by [16], and in fact, this is equivalent to having two fluids of equal permittivity. The superscripts $+$ and − are used to denote variables in the upper and lower fluids, respectively, and the permittivities are denoted by $\epsilon _0\epsilon _{\pm }$ where $\epsilon _0$ is the permittivity of free space—hence, $\epsilon _\pm$ are dimensionless and in what follows the ratio $\epsilon =\epsilon _+/\epsilon _-$ will become important. We also note that this setup was introduced and studied by Melcher [5] in his seminal monograph that established the field of interfacial electrohydrodynamics (see also Melcher and Taylor, Saville reviews). Our study extends that of [5] by introducing a physical dispersive regularisation by the inclusion of surface tension—in the absence of a regularisation, the $2\times 2$ system of nonlinear model equations not only is at best hyperbolic, and hence terminates in shocks, but can also be elliptic (with catastrophic short-wave instabilities) if the electric field is sufficiently strong, a case that was not studied in [5]. Here, we present a complete study of the regularised system and in particular show the existence of solitary waves and construct families of them.

The fluids are irrotational and incompressible; hence, the velocity field can be written as $\varvec{u}^{\pm }=\nabla \phi ^{\pm }$ where $\phi ^\pm$ denote the velocity potential in each region. Incompressibility then implies that $\phi ^\pm$ are harmonic. The electrostatics is also governed by Laplace equations for $V^\pm$. To see this, start with the electrostatic limit of the Maxwell equations so that $\varvec{\nabla }\times \varvec{E}^\pm =0$, where $\varvec{E}$ is the electric field, introduce voltage potentials so that $\varvec{E}^\pm =-\nabla V^\pm$, and use Gauss’s law $\varvec{\nabla }\cdot (\epsilon _0\epsilon _\pm \,\varvec{E}^\pm )=0$ in each region to obtain the result (the permittivities are constant). The boundary conditions at the walls are fluid impermeability and fixed voltage potentials. Those at the interface $y=S(x,t)$ are the kinematic conditions, continuity of the voltage potential, continuity of the *electric displacement* deriving from Gauss’s law, and continuity of normal stresses (tangential stresses cannot be prescribed, since the fluids are inviscid—slip is allowed at the interface). Hence, the governing equations and boundary conditions are

2.1a $$\begin{aligned}&\nabla ^2 \phi ^{\pm }=0, \end{aligned}$$

2.1b $$\begin{aligned}&\nabla ^2 V^{\pm }=0, \end{aligned}$$

2.1c $$\begin{aligned}&\phi _y^-|_{y=-h}=\phi _y^+|_{y=h}=0,\quad V^-=V^+-V_0=0,\end{aligned}$$

2.1d $$\begin{aligned}&\phi _y^{\pm }=S_t+\phi _x^\pm \,S_x\quad \mathrm {at} \quad y=S,\end{aligned}$$

2.1e $$\begin{aligned}&V^{+}-V^-=0 \quad \mathrm {at} \quad y=S,\end{aligned}$$

2.1f $$\begin{aligned}&\epsilon ^+\,\varvec{n} \cdot \nabla V^+ -\epsilon ^-\,\varvec{n} \cdot \nabla V^-=0 \quad \mathrm {at} \quad y=S,\end{aligned}$$

2.1g $$\begin{aligned}&\varvec{n}^\top \underline{\underline{\mathbf {\mathcal {T}}}}^+\ \varvec{n}-\varvec{n}^\top \underline{\underline{\mathbf {\mathcal {T}}}}^-\ \varvec{n}= -\sigma \kappa \quad \mathrm {at} \quad y=S. \end{aligned}$$

In (2.1f), (2.1g), $\top$ is the transpose, $\varvec{n}=(-S_x, 1)/\sqrt{1+S_x^2}$ is the unit normal pointing into fluid $+$, and $\underline{\underline{\mathbf {\mathcal {T}}}}$ is the combined fluid and Maxwell stress tensor which reads

2.2 $$\begin{aligned} \mathcal {T}_{ij}^\pm =-p^\pm \delta _{ij}+\epsilon _0\epsilon _\pm \left( E_i^\pm E_j^\pm -\frac{1}{2}|\mathbf {E}^\pm |^2\,\delta _{ij}\right) . \end{aligned}$$

The parameter $\sigma$ is the constant surface tension between the fluids, and $\kappa =S_{xx}/(1+S_x^2)^{3/2}$ is the signed curvature of the interface. Imposition of initial conditions completes the mathematical statement of the problem.

We proceed to non-dimensionalise the problem taking a typical interfacial wavelength to be *L*, and a typical dimensional speed to be $\overline{c}$. The interface is scaled by the channel height *h* and the pressure by its inertial scale. We write

2.3 $$\begin{aligned} x=LX^*,\quad y=hY^*, \quad S=hS^*, \quad t=\frac{2L}{\overline{c}}T^*,\quad \phi = \overline{c} L \Phi ^*, \quad V=\overline{V}_0 V^*, \quad p=\frac{\rho ^- (\overline{c})^2}{2} P^*, \end{aligned}$$

where starred variables are dimensionless. It is convenient to introduce the following dimensionless parameters that arise from the scalings (2.3):

2.4 $$\begin{aligned} \beta = \frac{h}{L}, \quad \rho =\frac{\rho ^+}{\rho ^-}, \quad F=\frac{\overline{c}^2}{2gh}, \quad \sigma ^*=\frac{2\sigma \beta }{L \rho ^- \overline{c}^2},\quad E_b=\frac{2 \epsilon _0\overline{V}_0^2}{\rho ^-\, \overline{c}^2\,h^2}. \end{aligned}$$

These can be identified as a slenderness parameter $\beta$, a density ratio $\rho$, a Froude number *F* measuring the importance of gravity, a scaled inverse Weber number $\sigma ^*$ that we retain in order to keep surface tension, and an electric Weber number measuring the ratio of electrostatic to inertial pressures. The scaled inverse Weber number $\sigma ^*$ is taken to be an $\mathcal {O}\big (\frac{1}{\beta }\big )$ parameter to allow the surface tension to compete with gravity and the electric field. In what follows we also use the Atwood ratio:

2.5 $$\begin{aligned} \alpha =\frac{1-\rho }{1+\rho }. \end{aligned}$$

Dropping the $*$ from our dimensionless variables, we write the governing equations as follows:

2.6a $$\begin{aligned}&\beta ^2 \Phi _{XX}^{\pm } + \Phi ^{\pm }_{YY}=0, \end{aligned}$$

2.6b $$\begin{aligned}&\beta ^2 V^{\pm }_{XX}+ V^{\pm }_{YY}=0, \end{aligned}$$

with the boundary conditions for $\Phi ^\pm$ at the interface taking the form:

2.7a $$\begin{aligned}&\Phi ^{\pm }_Y = \beta ^2 \left( \frac{1}{2} S_T + \Phi _X^{\pm } S_X \right) \quad \mathrm {at} \ Y=S, \end{aligned}$$

2.7b $$\begin{aligned}&\Phi ^-_T + \left( \Phi ^-_X\right) ^2 + \frac{1}{\beta ^2} \left( \Phi ^-_Y\right) ^2 - \rho \left( \Phi ^+_T + \left( \Phi ^+_X\right) ^2 + \frac{1}{\beta ^2} \left( \Phi ^+_Y\right) ^2 \right) +\frac{1- \rho }{F} S= P^+- P^- \ \ \mathrm {at} \ Y=S. \end{aligned}$$

Equation (2.7b) is the Bernoulli equation at the interface and is derived in the familiar way of integrating the Euler equations to obtain

2.8 $$\begin{aligned} \Phi _t^{\pm }+\left( \left( \Phi ^\pm _X\right) ^2+\frac{1}{\beta ^2}\left( \Phi ^\pm _Y\right) ^2\right) +Y=-\frac{\rho ^-}{\rho ^{\pm }}P^\pm +\mathrm {constant} \end{aligned}$$

and evaluating this equation at each side of the interface—see [8] for its use in electrohydrodynamic problems.

The voltage potential boundary conditions at $Y=S(X,T)$ become

2.9a $$\begin{aligned}&V^+-V^- =0, \end{aligned}$$

2.9b $$\begin{aligned}&\epsilon _+\left( V^{+}_Y- \beta ^{2} S_X V_X^{ +}\right) = \epsilon _-\left( V_Y^{ - } - \beta ^2 S_X V_X^{-}\right) , \end{aligned}$$

while the conditions at the walls yield

2.10a $$\begin{aligned}&\Phi ^{-}_Y|_{Y=-1}=0,\quad \Phi _Y^{+}|_{Y=1}=0, \end{aligned}$$

2.10b $$\begin{aligned}&V^{-}|_{Y=-1}=0, \quad V^{+}|_{Y=1}=1. \end{aligned}$$

Using the expression (2.2) in the normal stress balance (2.1g) and non-dimensionalising as described above, yields the following expression for the pressure jump across the interface

2.11 $$\begin{aligned} P^+-P^-= & {} \frac{\sigma S_{XX}}{(1+\beta ^2 S_X^{2})^{\frac{3}{2}}} - \frac{2 E_b\epsilon _+ \beta ^2 V_X^+ V_Y^+ S_X}{1+\beta ^2 S^{2}_X} + \frac{2 E_b \epsilon _- \beta ^2 V_X^- V_Y^- S_X}{1+\beta ^2 S_X^{2}}\nonumber \\&+\frac{E_b}{2} \frac{\beta ^2 S_X^{2} -1}{1+\beta ^2 S_X^{2}} \left[ \epsilon _+\left( \beta ^2(V_X^{+})^2-(V_Y^{+})^2\right) - \epsilon _- \left( \beta ^2(V_X^{-})^2-(V_Y^{-})^2 \right) \right] , \end{aligned}$$

to be used in the Bernoulli equation (2.7b), thus, eliminating the pressure. Our system of equations at this point is still exact, and a numerical procedure based on the full equations is necessary. In what follows we consider the long wave limit of $\beta \ll 1$ and make progress asymptotically to derive reduced-dimension nonlinear evolution equations for the flow.

## Asymptotic expansions and derivation of the evolution equations

In the limit $\beta \rightarrow 0$, we seek a solution of the problem by the asymptotic expansions

3.1a $$\begin{aligned}&V^{\pm }=V_0^{\pm }+ \beta ^2 V_1^{\pm }+\cdots , \end{aligned}$$

3.1b $$\begin{aligned}&\Phi ^{\pm }=\Phi _0^{\pm }+ \beta ^2 \Phi _1^{\pm }+\cdots ,\end{aligned}$$

3.1c $$\begin{aligned}&S=S_0+\beta ^2 S_1+\cdots . \end{aligned}$$

These expansions are valid as long as gradients, e.g. $S_{0x}$, remain bounded, and this is confirmed *a posteriori* by solving the resulting equations. Substituting expansions () into the dimensionless system (), we obtain to leading order

3.2 $$\begin{aligned} \Phi ^{\pm }_{0YY}=0, \quad V^{\pm }_{0YY}=0, \end{aligned}$$

which can be readily integrated to give

3.3 $$\begin{aligned} \Phi _0^\pm =A_1^{\pm }(X,T)\,Y+A_2^\pm (X,T),\quad V_0^\pm =B_1^{\pm }(X,T)\,Y+B_2^\pm (X,T), \end{aligned}$$

where the functions $A_{1,2}^\pm$ and $B_{1,2}^\pm$ must be determined. Using the no penetration conditions (2.10a) at the walls immediately implies that $A_1^\pm \equiv 0$, and hence, $\Phi _0^\pm \equiv \Phi _0^\pm (X,T)$, i.e. they are independent of *Y*. Equation (2.6a) at order $\beta ^2$ gives

3.4 $$\begin{aligned} \Phi _{1YY}^\pm =-\Phi _{0XX}^\pm , \end{aligned}$$

which when integrated once and on use of the no penetration wall conditions yields

3.5 $$\begin{aligned} \Phi _{1Y}^-=-(Y+1)\,\Phi _{0XX}^-,\quad \Phi _{1Y}^+=(1-Y)\,\Phi _{0XX}^+. \end{aligned}$$

These expressions will be useful in the first-order kinematic conditions considered below.

The wall voltage conditions (2.10b) yield, in turn,

3.6 $$\begin{aligned} V_0^-=(Y+1)\,B_2^-(X,T),\quad V_0^+=(1-Y)\, B_2^+(X,T). \end{aligned}$$

The unknown functions in (3.6) can be found from the leading order contributions to the voltage and displacement field continuity at the interface equations: (2.9a)–(2.9b), i.e. $V_0^-=V_0^+$ and $\epsilon _- V_{0Y}^-=\epsilon _+V_{0Y}^+$ at $Y=S_0(X,T)$, to give

3.7 $$\begin{aligned} V^+_0=\frac{Y-1}{\epsilon + 1 + S_0(\epsilon -1)}+1, \quad V_0^-=\frac{\epsilon (1+Y)}{\epsilon + 1 + S_0(\epsilon -1)}. \end{aligned}$$

In order to obtain equations for the remaining unknowns $S_0$ and $\Phi _0^{\pm }$, we use an approach similar to [16]. From the $\mathcal {O}(\beta ^2)$ kinematic conditions (2.7a) on either side of the interface, and using the solutions (3.5), we find

3.8a $$\begin{aligned}&\frac{1}{2} S_{0T}+\left( (S_0+1)\Phi _{0X}^-\right) _X=0, \end{aligned}$$

3.8b $$\begin{aligned}&\frac{1}{2} S_{0T}+\left( (S_0-1)\Phi _{0X}^+\right) _X=0. \end{aligned}$$

We now define new variables, *U* and *W*, representing the average horizontal velocity and half its jump across the interface,

3.9 $$\begin{aligned} U=\frac{1}{2}\left( \Phi _{0X}^++\Phi _{0X}^-\right) ,\quad W=\frac{1}{2}\left( \Phi ^+_{0X}-\Phi ^-_{0X}\right) . \end{aligned}$$

Subtracting either of the equations in (3.8) from the other and integrating with respect to *X* gives

3.10 $$\begin{aligned} U-S_0 W =\chi (T), \end{aligned}$$

where $\chi (T)$ is an arbitrary function of *T*. Adding equations (3.8a) and (3.8b) and using (3.10) to eliminate *U* yield

3.11 $$\begin{aligned} S_{0T}+2\chi \, S_{0X} +2\left( \left( S_0^2-1\right) W\right) _X=0. \end{aligned}$$

A second-evolution equation follows from the Bernoulli equation (2.7b) after its differentiation with respect to *X* and use of (3.9) and (3.10). The result is (recall that $\alpha$ is the Atwood number defined in (2.5))

3.12 $$\begin{aligned}&\left( W-\alpha S_0 W\right) _T+2\chi (T)\left( W-\alpha S_0 W\right) _X -\left( \alpha S_0^2 W^2 -2S_0W^2 + \alpha W^2\right) _X\nonumber \\&\quad = \frac{\alpha }{F} S_{0X} - \frac{\sigma }{1+\rho }S_{0XXX} - \frac{E_b}{2(1+\rho )}\left[ \epsilon _+ \left( V^+_{0Y}\right) ^2- \epsilon _-\left( V^-_{0Y}\right) ^2\right] _X. \end{aligned}$$

Changing to the moving frame $(X,T)\rightarrow (X-2 \int \chi (T) \mathrm{{d}}T, T)$ eliminates $\chi (T)$ terms, so up to $\mathcal {O}(\beta )$, $\mathcal {O}(\beta ^2)$ respectively the coupled system of equations are (we use *X* for the new spatial variable and drop the subscript 0 from $S_0, V_0$)

3.13a $$\begin{aligned}&S_{T} +2(S^2 W)_X=2W_{X}, \end{aligned}$$

3.13b $$\begin{aligned}&\left( W-\alpha S W\right) _T -\left( \alpha S^2 W^2 -2SW^2 + \alpha W^2\right) _X = \frac{\alpha }{F} S_X - \frac{\sigma }{1+\rho }S_{XXX} - \frac{E_b}{2(1+\rho )}\left[ \epsilon _+ \left( V^+_{Y}\right) ^2- \epsilon _-\left( V^-_{Y}\right) ^2\right] _X.\nonumber \\ \end{aligned}$$

Defining a new variable

3.14 $$\begin{aligned} \Delta = W(1-\alpha S), \end{aligned}$$

allows us to write () as the following dispersively modified $2\times 2$ system of conservation laws

3.15 $$\begin{aligned} \begin{pmatrix} S \\ \Delta \end{pmatrix}_T+ \begin{pmatrix} Q_1 &{}\quad Q_2 \\ Q_3 &{}\quad Q_4 \end{pmatrix} \begin{pmatrix} S \\ \Delta \end{pmatrix}_\chi = \begin{pmatrix} 0 \\ -\frac{\sigma }{1+\rho }S_{XXX} \end{pmatrix}, \end{aligned}$$

where the matrix entries are

3.16a $$\begin{aligned}&Q_1= \frac{2 \Delta }{(1- \alpha S)^2}\left( 2S(1-\alpha S) +\alpha (S^2-1) \right) , \end{aligned}$$

3.16b $$\begin{aligned}&Q_2= 2\frac{S^2-1}{1- \alpha S}, \end{aligned}$$

3.16c $$\begin{aligned}&Q_3= - \left( \frac{\alpha }{F}+ \frac{2( \alpha S-1) \Delta ^2}{(1-\alpha S)^2}+ \Delta ^2(\alpha S^2 + \alpha -2S) \frac{2 \alpha }{(1-\alpha S)^3} - \frac{E_b \epsilon _+}{2(1+\rho )} \frac{(1-\epsilon )^2}{(\epsilon +1+S(\epsilon -1))^3} \right) ,\end{aligned}$$

3.16d $$\begin{aligned}&Q_4=- 2\left( \frac{\alpha S^2 \Delta }{(1-\alpha S)^2}+ \frac{\Delta (\alpha -2S)}{(1-\alpha S)^2} \right) . \end{aligned}$$

As we will see below, the electric field has a considerable effect on the flow by introducing complex eigenvalues for the matrix *Q*. In the absence of an electric field, most easily recovered by setting $\epsilon =1$ in (3.15), we recover the equations of [16] where the eigenvalues of the analogous matrix *Q* are now real, and hence, in the absence of dispersion, the system is hyperbolic.

It is instructive to consider the linear limit of this problem for expanding *S*, $\Delta$ like3.17where $\zeta \ll 1$. The system (3.15) can be reduced to a single equation for $\tilde{S}_0$

3.18 $$\begin{aligned} \frac{1}{2}\left( \tilde{S}_0\right) _{TT}=\left( \frac{\alpha }{F}- \frac{E_b \epsilon _+}{2(1+\rho )} \frac{(1-\epsilon )^2}{(\epsilon +1)^3} \right) (\tilde{S}_0)_{XX}-\frac{\sigma }{1+\rho }(\tilde{S}_0)_{XXXX}. \end{aligned}$$

Looking for a wave-like solution of the form $\tilde{S_0}=e^{ik X+\omega t}\hat{S}(X, T)$ gives the dispersion relation

3.19 $$\begin{aligned} \omega ^2=-2k^2\left( \frac{\sigma }{1+\rho }k^2+\frac{\alpha }{F}- \frac{E_b \epsilon _+}{2(1+\rho )} \frac{(1-\epsilon )^2}{(\epsilon +1)^3} \right) . \end{aligned}$$

Therefore, if

3.20 $$\begin{aligned} \frac{\alpha }{F}- \frac{E_b \epsilon _+}{2(1+\rho )} \frac{(1-\epsilon )^2}{(\epsilon +1)^3}<0 \end{aligned}$$

we have growth, and if

3.21 $$\begin{aligned} \frac{\alpha }{F}- \frac{E_b \epsilon _+}{2(1+\rho )} \frac{(1-\epsilon )^2}{(\epsilon +1)^3}\ge 0 \end{aligned}$$

the problem is dispersive, so under no circumstances is the linear system dissipative.

The full problem stated in (3.15) is a nonlinear free boundary one with two fluid phases and a large number of physical parameters. The asymptotic reduction supports analytical progress and in particular allows for the exploration of nonlinear coherent structures such as travelling wave trains and solitary waves. We explore such possibilities next.

## Solitary waves: existence and construction

### Solitary waves at arbitrary density ratios

We will construct solitary wave solutions of the system (3.13) and find the range of parameters for which they exist. Looking for travelling wave solutions of () of the form

4.1 $$\begin{aligned} S=S(\xi ),\quad W=W(\xi ),\quad \xi =X+cT, \end{aligned}$$

where *c* is the positive wavespeed, yields the coupled ordinary differential equations

4.2a $$\begin{aligned}&-\,cS_\xi +2(S^2W)_\xi =2W_\xi , \end{aligned}$$

4.2b $$\begin{aligned}&-\,c(W-\alpha S W)_\xi -(\alpha S^2 W^2 -2SW^2 + \alpha W^2)_\xi \nonumber \\&\quad =\frac{\alpha }{F} S_\xi - \frac{\sigma }{1+\rho }S_{\xi \xi \xi } - \frac{E_b}{2(1+\rho )}\left[ \epsilon _+ \left( V^+_{Y}\right) ^2- \epsilon _-\left( V^-_{Y}\right) ^2\right] _\xi . \end{aligned}$$

where *c* is the wave-speed. It is trivial to solve the first equation for *W* in terms of *S*, thereby eliminating *W* from the problem; we find

4.3 $$\begin{aligned} W=-\frac{1}{2} \frac{D+cS}{1-S^2}, \end{aligned}$$

where *D* is a constant of integration. In order to integrate (4.2b), we will need

4.4 $$\begin{aligned} \int \left[ \epsilon _+ \left( V^+_{Y}\right) ^2- \epsilon _-\left( V^-_{Y}\right) ^2\right] \mathrm{{d}}S= \frac{\epsilon _+}{\epsilon +1 +S(\epsilon -1)}+C_1, \end{aligned}$$

where $C_1$ is a constant-this follows by use of the expressions (4.3). Next, we integrate (4.2b) with respect to $\xi$, multiply the result by $S_\xi$, carry out an additional $\xi$ integration using (4.4), and yield the following dynamical system for *S*:

4.5 $$\begin{aligned} \frac{1}{2} \gamma (S_\xi )^2&= \frac{\alpha }{2F} S^2 +GS-\frac{1}{4}\alpha c^2 S+ H -\frac{1}{8} \frac{(D+c)^2(1-\alpha )}{1-S}\nonumber \\&\quad -\frac{1}{8} \frac{(D-c)^2(1+\alpha )}{1+S} -\frac{\delta }{2} \frac{1}{\epsilon +1+S(\epsilon -1)}, \end{aligned}$$

where *G* and *H* are constants and the parameters

4.6 $$\begin{aligned} \gamma =\frac{\sigma }{1+\rho }>0,\quad \delta =\frac{E_b \epsilon _+}{1+\rho }, \end{aligned}$$

measure surface tension and electric field strength, noting that $\gamma >0$ and $\delta \ge 0$. By requiring that as $|\xi | \rightarrow \infty$, *S* and all its derivatives tend to 0 (thus restricting our solutions to solitary waves), we see from (4.3) that *D* is the jump in horizontal velocity across the interface, i.e. it is the undisturbed vortex sheet strength. Equation (4.5) becomes, after appropriate selection of *G* and *H* to achieve the desired decay at infinity,

4.7 $$\begin{aligned} \gamma (S_\xi )^2=S^2 \left[ \frac{\alpha }{F} + \frac{\alpha S -1}{2(1-S^2)}\left( D^2+c^2-2cD \frac{S-\alpha }{\alpha S -1}\right) \right] - \delta \frac{S^2(\epsilon -1)^2}{(\epsilon +1)^2(\epsilon +1+S(\epsilon -1))}. \end{aligned}$$

Note that for solitary waves, $\gamma$ can be scaled to unity by a redefinition of $\xi$. We can see that this equation implies that $\alpha >0$ is a necessary condition for solitary waves to exist, hence excluding Rayleigh–Taylor unstable configurations as would be expected. Note that this condition is the same as in the non-electrified case considered by [16], consistent from the fact that a vertical electric field is destabilising. In order to analyse the properties of the solitary waves that may be present in the channel, it is useful to rewrite Eq. (4.7) in the form

4.8 $$\begin{aligned} \gamma (S_\xi )^2=\frac{S^2}{(1-S^2)\left[ \epsilon +1 +S(\epsilon -1)\right] }\,p_3(S), \end{aligned}$$

where $p_3(S)$ is a cubic polynomial given by

4.9 $$\begin{aligned} p_3(S)=jS^3+kS^2 + lS+m. \end{aligned}$$

The constants *j*, *k*, *l* and *m* are given by

4.10a $$\begin{aligned}&j=\frac{\alpha }{F}(1-\epsilon ), \end{aligned}$$

4.10b $$\begin{aligned}&k=\delta \frac{(\epsilon -1)^2}{(\epsilon +1)^2} +\alpha \left[ -\frac{\epsilon +1}{F} + \frac{1}{2}(\epsilon -1)\left( D^2+c^2-\frac{2cD}{\alpha }\right) \right] ,\end{aligned}$$

4.10c $$\begin{aligned}&l=\frac{\alpha }{2}(\epsilon +1)\left( D^2+c^2-\frac{2cD}{\alpha }\right) +(\epsilon -1)\left( \frac{\alpha }{F}-\frac{D^2+c^2}{2}+cD\alpha \right) ,\end{aligned}$$

4.10d $$\begin{aligned}&m=(\epsilon +1)\left( \frac{\alpha }{F}- \frac{D^2+c^2}{2} +\alpha D c \right) - \delta \frac{(\epsilon -1)^2}{(\epsilon +1)^2}. \end{aligned}$$

Inspection of (4.8) and noting the facts $|S|<1$ and $\epsilon +1+S(\epsilon -1)>0$, implies that the sign of $p_3$ determines the existence of solitary waves. First, we must have $p_3(S)>0$ for small |*S*| so that $S_\xi ^2$ is positive. By continuity, we require $p_3(0)=m>0$ which gives the following necessary condition for the existence of solitary waves:

4.11 $$\begin{aligned} (\epsilon +1)\left( \frac{\alpha }{F}- \frac{D^2+c^2}{2} +\alpha Dc \right) - \delta \frac{(\epsilon -1)^2}{(\epsilon +1)^2}>0. \end{aligned}$$

Solitary waves emerge when we can connect $S=0$ to another root(s) $S_r$, say, as long as $|S_r|<1$. For this to happen, we need at least one real root of $p_3(S)$ to lie in $-1<S<1$. Now,

4.12 $$\begin{aligned} p_3(-1)=-(\alpha +1)(D-c)^2< 0, \quad p_3(1)=\epsilon (\alpha -1)(D+c)^2< 0, \end{aligned}$$

with $p_3(1)=0$ when $\alpha =1$, i.e. $\rho =0$. This is possible when the upper fluid is passive – this is considered in more detail later—see also [16]. Further special cases arise when $D=\pm c$, in which case $p_3$ has roots at either $S=-1$ or $S=1$, and in both cases, it is possible to have zero, one, or two solitary waves produced depending on the other parameters—this has been confirmed numerically, but details are not included for brevity. As these cases do not correspond to a meaningful physical limit since the interface touches the wall, they have not been investigated further.

**Fig. 2:**
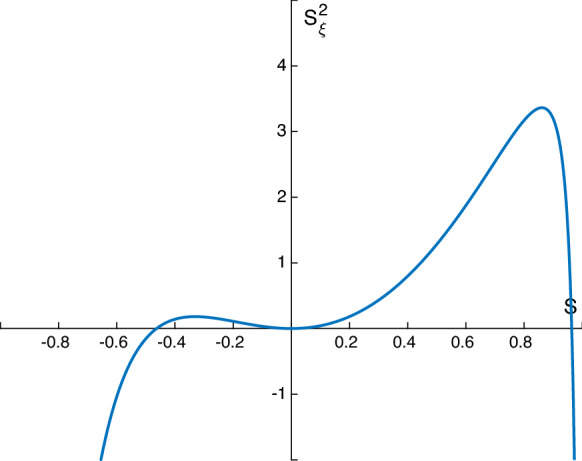
Sketch of the admissible depression and elevation solitary wave trajectories in the phase plane of the dynamical system in the range $-1<S<1$

From (4.12), we conclude that if it is assumed that $p_3(S)$ has real roots in $-1<S<1$ (something that is necessary for solitary waves to exist), then it must have exactly two roots or a repeated double root; otherwise, it is impossible to satisfy all the inequalities of (4.11), (4.12). The latter scenario is inadmissible because it forces $S_\xi ^2<0$ in the vicinity of $S=0$ even if the repeated root is at $S=0$. The former scenario is again inadmissible unless the roots have opposite signs, and this is the generic situation for the existence of solitary waves in this rather general case. Interestingly, we establish that solitary waves come in pairs, an elevation wave corresponding to $1>S_r^+>0$ and a depression (or dark) solitary wave for the root $-1<S_r^-<0$. An illustration of this is provided in Fig. 2, in which regions where $(S_\xi )^2<0$ are shown for mathematical completeness despite this not being physical. Integration of (4.8) provides integrals for the solutions analogous to (4.19) constructed below.

### Solitary waves with upper fluid of zero density ($\alpha =1$)

When the upper fluid has negligible density compared to the lower fluid the Atwood ratio $\alpha =1$. The equations simplify in this case, and in particular are not prone to a Kelvin–Helmholtz instability since the system is one-sided for the hydrodynamics. We begin with a general dielectric fluid in the lower region and then consider the additional limit of a perfectly conducting lower liquid first studied by Melcher [5] (Chapter 7).

#### Perfect dielectric lower fluid

In order for this model to be physical we will assume that the ratio of permittivities is $\epsilon <1$. In this special case, $S=1$ is a root of $p_3$ so we can simplify (4.8) to

4.13 $$\begin{aligned} \gamma (S_\xi )^2=\frac{S^2}{(1+S)(\epsilon +1+S(\epsilon -1))} p_2(S), \end{aligned}$$

where

4.14 $$\begin{aligned} p_2(S)&=S^2 \frac{1}{F}(\epsilon -1)+S \left( \frac{2\epsilon }{F}-\delta \frac{(\epsilon -1)^2}{(\epsilon +1)^2} -\frac{1}{2}(\epsilon -1)(D-c)^2 \right) \nonumber \\&\quad - \,\delta \frac{(\epsilon -1)^2}{(\epsilon +1)^2}+\frac{1}{F}(\epsilon +1)-\frac{1}{2}(\epsilon +1)(D-c)^2. \end{aligned}$$

Evaluating $p_2$ at $S=0$ and requiring $p_2(0)>0$, it can be seen that in this case, the necessary condition for solitary waves to be produced is

4.15 $$\begin{aligned} \frac{2}{F}-2\delta \frac{(\epsilon -1)^2}{(\epsilon +1)^3}-(D-c)^2>0. \end{aligned}$$

**Fig. 3:**
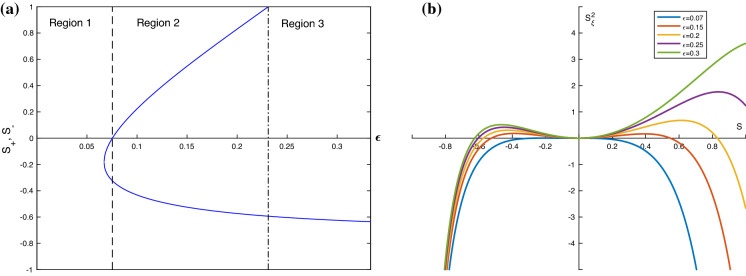
Dependence of the number of roots on $\epsilon$, with $\alpha =1$, with $c=2.5, \ \sigma =1, \ F=0.1, \ \delta =10, \ D=0.$

As in the previous section, we have that $p_2(-1)=-(D-c)^2<0$, so for the existence of solitary waves, we require one root of $p_2$ is $-1<S<0$. However, the right-hand side of (4.13) is now finite as $S\rightarrow 1$ and can take either a positive or negative value. Assuming that there is one root of $p_2$ in the range $-1<S<0$, if the second root is in $0<S<1$, then there will be a second solitary wave produced. This extra condition is equivalent to requiring $p_2(1)=\frac{4\epsilon }{F}-\frac{2\delta (\epsilon -1)^2}{(\epsilon +1)^2}-\epsilon (D-c)^2<0$. So given that there is one root in $-1<S<0$, if $p_2(1)$ is positive we have only one depression solitary wave, if it is negative, we have an additional elevation solitary wave. An example of how the value of $p_2(1)$ depends on $\epsilon$ is given in Fig. 3b, with $\epsilon = 0.15\, 0.2$ giving $p_2(1)<1$ so producing two solitary waves. From (4.15) and the expression for $p_2(1)$, we have the explicit condition for the existence of two solitary waves:

4.16 $$\begin{aligned} \frac{4}{F}-2\delta \frac{(\epsilon -1)^2}{\epsilon (\epsilon +1)^2}<(D-c)^2<\frac{2}{F}-2\delta \frac{(\epsilon -1)^2}{(\epsilon +1)^3}. \end{aligned}$$

For one solitary wave, we must have

4.17 $$\begin{aligned} (D-c)^2< \frac{2}{F}-2\delta \frac{(\epsilon -1)^2}{(\epsilon +1)^3}\quad \mathrm{and}\quad (D-c)^2<\frac{4}{F}-2\delta \frac{(\epsilon -1)^2}{\epsilon (\epsilon +1)^2}. \end{aligned}$$

The dependence on the number of roots on the ratio of permitivitties $\epsilon$ can be seen in Fig. 3a for a typical set of parameter values given in the caption. These can be found explicitly from (4.14), and they are plotted when they are real as functions of $\epsilon$. In region 1, there are no admissible solitary waves since the roots are both negative. For $\epsilon >0.07$ we have one positive and one negative root, admitting two solitary waves. Region 2 terminates at $\epsilon \approx 0.23$ since the positive root now equals unity, i.e. touches the wall and must be excluded. Beyond this value we have region 3 which supports a single depression solitary wave. The region 3 solutions are consistent with the results of [16] who found only depression solitary waves in the case of no electric field equivalent to the $\epsilon =1$ case here. Example phase planes for certain values of $\epsilon$ are given in Fig. 3b.

We can also numerically solve Eq. (4.13) for *S* and construct solitary waves. In the present case, we have either one or two roots of $p_2(S)$ in the physical range $-1<S<1$. As discussed above, we require exactly one root, denoted by $S_-$, to be in the range $-1<S_-<0$. Denoting the other root of $p_2(S)$ to be $S_0$ (note that we do not assume $0<S_0<1$), we can write (4.13) in the form

4.18 $$\begin{aligned} \gamma (S_\xi )^2=\left( \frac{\epsilon -1}{F}\right) \frac{S^2(S-S_-)(S-S_0)}{(1+S)(1+\epsilon +S(\epsilon -1))}. \end{aligned}$$

Separating variables and integrating from the trough $S=S_-$ to an elevation height *S* yields

4.19 $$\begin{aligned} \int _{S_-}^S\left[ \left( \frac{F}{\epsilon -1}\right) \frac{(1+z)(1+\epsilon +z(\epsilon -1))}{(z-S_-)(z-S_0)}\right] ^{\frac{1}{2}} \frac{\mathrm{{d}}z}{z}=\frac{\xi }{\sqrt{\gamma }}. \end{aligned}$$

**Fig. 4:**
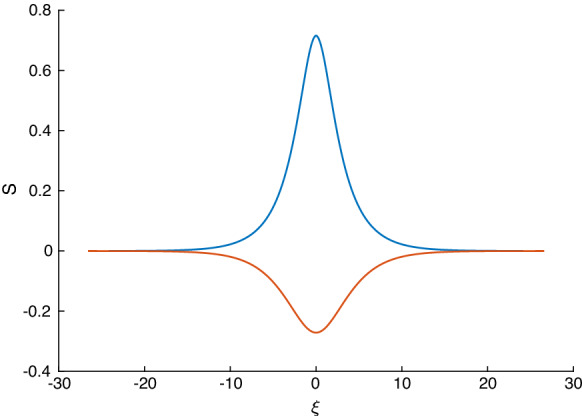
Example of a pair of solitary waves when $\alpha =1$, with $c=\sigma =F=\delta =1, \ D=0, \ \epsilon =\frac{1}{5}$

The positive square root was taken in (4.19) to produce half the wave—the negative root gives the other symmetric half. Numerically solving this equation for varying parameters produces the lower (red) solitary wave depicted in Fig. 4. A depression solitary wave is always produced if the necessary condition (4.15) for solitary waves is met. If in addition (4.16) is met so that $0<S_0<1$, then a second elevation solitary wave is supported having the same speed as its depression counterpart. In the example of Fig. 4 we also show the elevation wave in blue.

To understand the mathematical structure of the constructed solitary waves we consider the evolution PDEs (3.15) with $\alpha =1$. (Note that in general (3.15) recover the equations in [5] when $\sigma =0$.) The equations are

4.20 $$\begin{aligned} \begin{pmatrix} S \\ \Delta \end{pmatrix}_T+ \begin{pmatrix} -2 \Delta &{} -2(1+S) \\ - \frac{1}{F} +2 \delta \frac{(1-\epsilon )^2}{(\epsilon +1+S(\epsilon -1))^3} &{} - 2 \Delta \end{pmatrix} \begin{pmatrix} S \\ \Delta \end{pmatrix}_X=\begin{pmatrix} 0 \\ -\sigma S_{XXX}, \end{pmatrix} \end{aligned}$$

where in this case, we have that for solitary waves,

4.21 $$\begin{aligned} \Delta =-\frac{1}{2} \left( \frac{D+cS}{1+S}\right) . \end{aligned}$$

If the eigenvalues of the nonlinear flux matrix in (4.20) are denoted by $\mu (S, \Delta )$, then we find that they are determined through the equation:

4.22 $$\begin{aligned} (\mu +2\Delta )^2 +2(1+S)\left[ \frac{2\delta (1-\epsilon )^2}{(\epsilon +1+S(\epsilon -1))^3}-\frac{1}{F}\right] =0. \end{aligned}$$

We find that complex eigenvalues of the flux matrix in (4.20) arise when

4.23 $$\begin{aligned} S>\frac{1+\epsilon -\left[ 2\delta F (1-\epsilon )^2\right] ^{1/3}}{1-\epsilon }:=S_{tr}. \end{aligned}$$

When we have $\mu$ is complex for a range of *S* we call such a region elliptic, and when $\mu \in \Re$, we refer to the region as hyperbolic, so $S_{tr}$ is the transition point where the system changes from hyperbolic to elliptic or vice versa. An immediate consequence of (4.23) is that when $\epsilon$ is near 1 we can make $S_{tr}>1$, i.e. elevation waves can be found which are what we term hyperbolic; this is in contrast to the $\epsilon =0$ case discussed in Sect. 4.2.2. We also note that the bound (4.23) only makes physical sense when the parameters are such that additionally $|S|<1$. In the case of two solitary waves, we have from the inequality: (4.16)

4.24 $$\begin{aligned} \frac{\epsilon (\epsilon +1)^3}{(\epsilon -1)^2}<\delta F < \frac{(\epsilon +1)^3}{(\epsilon -1)^2}. \end{aligned}$$

Using (4.24) in (4.23) provides the following range of values for the transition boundary $S=S_{tr}$:

4.25 $$\begin{aligned} \frac{(1-2^{1/3})(1+\epsilon )}{1-\epsilon }<S_{tr}<\frac{(1+\epsilon )(1-2^{1/3}\epsilon ^{1/3})}{1-\epsilon }. \end{aligned}$$

This inequality in turn predicts that the solitary waves produced when $\alpha =1$, $0<\epsilon <1$, can potentially have parts where the equations are locally elliptic and other parts where they are locally hyperbolic, in addition to wholly elliptic or hyperbolic. It is quite easy to check numerically the upper and lower bounds of $S_{tr}$ in (4.25) for $0<\epsilon <1$, and to confirm that transitions occur for both depression and elevation waves when $0<\epsilon \lessapprox 0.587$. Such analytical estimates were used prior to searching for transitional solitary waves like those in Fig. 6.

We note that the classification of solitary waves into elliptic and hyperbolic regions presented here does not take into account the dispersive term on the right-hand side of (4.20) so strictly speaking only concerns the conservation laws when $\sigma =0$. However, this diagnostic tool yields useful information because a change from real to complex eigenvalues of the conservation laws predicts the presence of instabilities that destroy the solitary wave structures, which enables us to predict instability of nonlinear solitary waves without doing any spectral analysis. Analogous analyses and classifications have been used in related viscous multifluid interfacial problems where the regularising terms are diffusive - see for example [17, 18].

#### Lower fluid a perfect conductor

As mentioned earlier this limit was considered in [5] in the absence of surface tension, and hence the solutions there cannot produce solitary waves. The perfect conductor lower fluid limit is found by sending its permittivity to infinity, $\epsilon _-\rightarrow \infty$. Hence, we have $\epsilon =\epsilon _+/\epsilon _-=0$ and Eq. (4.13) reduces to

4.26 $$\begin{aligned} \gamma (S_\xi )^2=\frac{S^2}{1-S^2}p_2^*(S), \end{aligned}$$

where $$p_2^*(S)$$ is

4.27 $$\begin{aligned} p_2^*(S)=-\frac{1}{F}S^2+S\left( \frac{1}{2}(D-c)^2 -\delta \right) -\delta +\frac{1}{F}-\frac{1}{2}(D-c)^2. \end{aligned}$$

Using similar reasoning as before, the necessary condition for solitary waves for this sub-case is $p_2^*(0)>0$

**Fig. 5:**
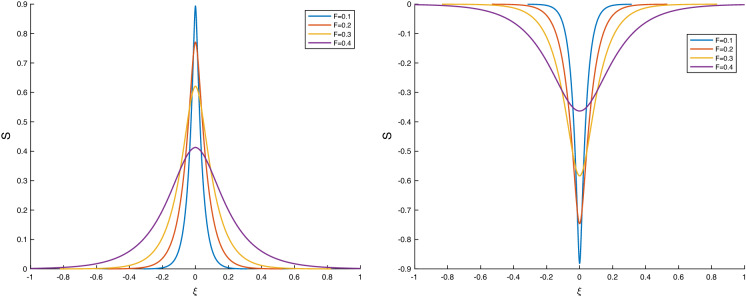
Pairs of solitary waves with $\epsilon =0,\ \alpha =1, \ \delta =1, \ \sigma =0.01, \ D=0, \ c=1.5$, and F varying

4.28 $$\begin{aligned} 0<(D-c)^2<\frac{2}{F}-2 \delta , \end{aligned}$$

and consequently solitary waves can only exist if $F<{1}/{\delta }$, i.e. for sufficiently small Froude numbers, given an electric field strength. We also note that $p_2^*(-1)=-(D-c)^2<0$, $p_2^*(1)=-2\delta <0$; hence, a solitary wave of depression and one of elevation coexist in general. Typical pairs of solitary waves are given in Fig. 5 as *F* varies.

For completeness, we consider the mathematical structure of the waves constructed in this section. The flux matrix in this case now reads

4.29 $$\begin{aligned} \begin{pmatrix} S \\ \Delta \end{pmatrix}_T+ \begin{pmatrix} -2 \Delta &{}\quad -2(1+S) \\ - \frac{1}{F} +2 \delta \frac{1}{(1-S)^3} &{}\quad - 2 \Delta \end{pmatrix} \begin{pmatrix} S \\ \Delta \end{pmatrix}_X=\begin{pmatrix} 0 \\ -\sigma S_{XXX} \end{pmatrix} \end{aligned}$$

and we note that for solitary waves (4.21) still holds. Repeating the hyperbolic-elliptic calculation of section 4.2.1, and now setting $\epsilon =0$, we find that complex eigenvalues of the flux matrix in (4.20) arise when

4.30 $$\begin{aligned} 2\delta F-(1-S)^3>0\quad \Rightarrow \quad S>1-(2\delta F)^{1/3}:=S_{a}. \end{aligned}$$

It can be shown that $S_a$ defined above is always smaller than the amplitude of elevation solitary waves given by the positive root of (4.27). From (4.28), we have $\delta F<1$; combining this with (4.30) and the physical fact that $\delta$ and *F* are non-negative, we arrive at the following general condition that is necessary for the evolution equations to become locally elliptic (we exclude the boundaries where (4.22) gives two equal and real eigenvalues)

4.31 $$\begin{aligned} 0<\delta F< 1. \end{aligned}$$

**Fig. 6:**
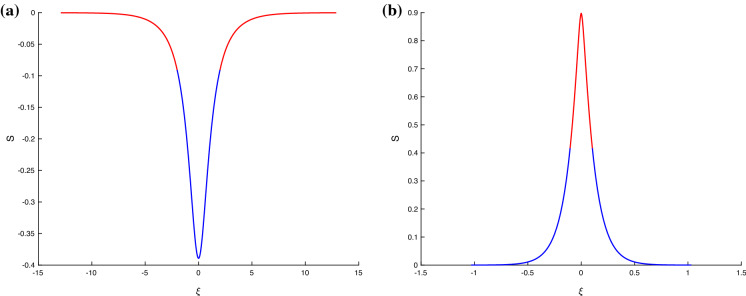
Solitary waves that transition from elliptic behaviour (red) to hyperbolic behaviour (blue). Here $\delta =1,\ \alpha =1,\ \epsilon =0, \ \sigma =0.1, \ D=0, \ c=1$. **a** $F=0.65$; **b** $F=0.1$. (Color figure online)

The inequality (4.30) in turn predicts that any solitary wave satisfying $1>S>1-2^{1/3}\approx -0.26$, can potentially have parts where the equations are locally elliptic and other parts where they are locally hyperbolic, in addition to wholly elliptic or hyperbolic. These properties hold for depression as well as elevation waves, and examples of such mixed behaviour are given in Fig. 6. Figure 6a has Froude number $F=0.65$, while Fig. 6b has $F=0.1$, the other parameters being $\delta =1$, $\sigma =0.1$ (note that $\alpha =1$ and $\epsilon =0$ here). For $F=0.65$, the depression wave exhibits mixed behaviour as seen in the figure that depicts the elliptic parts in red and the hyperbolic ones in blue. When $F=0.1$, the elevation wave now supports ellipticity where its amplitude is sufficiently large, as seen in Fig. 6b. We note that if transition takes place in the elevation wave then the depression wave is wholly hyperbolic, and if it takes place in the depression wave then the positive wave is wholly elliptic. This can be inferred from the monotonicity of the sufficient condition for ellipticity $1>S>1-2^{1/3}\approx -0.26$. Of course, exact diagnostics are calculated directly from the eigenvalues (4.22).

## Periodic travelling waves

Section 4 is concerned with solitary waves that decay far away. Here, we construct travelling waves of finite spatial period governed by equations (3.15). We will concentrate on the physical regime of Sect. 4.2.2, i.e. we take $\alpha =1$ and $\epsilon _-=\infty$ (i.e. $\epsilon =0$) which restricts our analysis to the case of a hydrodynamically passive upper dielectric region and a perfectly conducting lower fluid (the general case can be analysed in analogous ways and is excluded for brevity). Looking for travelling waves in a frame $\xi =X-cT$ as before, casts the system (3.15) into

5.1a $$\begin{aligned}&-\,cS_\xi -2\Delta S_\xi -2(1+S)\Delta _\xi =0, \end{aligned}$$

5.1b $$\begin{aligned}&-\,c\Delta _\xi + \left( - \frac{1}{F} + \delta \frac{1}{(1-S)^3} \right) S_\xi - 2 \Delta \Delta _\xi =-\sigma S_{\xi \xi \xi }. \end{aligned}$$

By inspection, solutions to this system can be even, and in what follows we consider solutions that are even about $\xi =0$ (Galilean invariance allows this). For periodic travelling waves, we further require that $S(L)=S(-L)$, $\Delta (L)=\Delta (-L)$ where 2*L* is the wavelength, and note that due to the assumed symmetry, we only need to solve the system on the half domain $0\le \xi \le L$.

For a given set of parameters $F,\ \sigma , \ \delta$, to solve (5.1), we require 4 boundary conditions, two of which we impose on *S* and two on $\Delta$, both at the points $\xi =0,\ L$. Integrating each of (5.1a) and (5.1b) between 0 and *L* yields the conditions

5.2a $$\begin{aligned} c= & {} 2\frac{(1+S(L))\Delta (L)-(1+S(0))\Delta (0)}{S(0)-S(L)}, \end{aligned}$$

5.2b $$\begin{aligned}&-\,\sigma (S_{\xi \xi }(L)-S_{\xi \xi }(0))= -c (\Delta (L)-\Delta (0)) -\frac{1}{F}(S(L)-S(0))\nonumber \\&+\,\frac{\delta }{2}\left( \frac{1}{(1-S(L))^2}-\frac{1}{(1-S(0))^2} \right) -(\Delta (L)^2-\Delta (0)^2), \end{aligned}$$

which can be thought of as two equations for the unknowns *c* and *L* for given physical parameters $F, \sigma , \delta$, and given wave and velocity amplitudes, i.e. the four values $S(0), S(L), \Delta (0), \Delta (L)$. Note that not all sets of parameters and end conditions give rise to physically admissible solutions. The integrals (5.2a)–(5.2b) allow elimination of *c* and derivation of a nonlinear relation between the end conditions which we conveniently state as the following quadratic equation for $\Delta (0)$:

5.3 $$\begin{aligned}&\Delta (0)^2 \Bigg (1+\frac{2(1+S(0))}{S(L)-S(0)}\Bigg )+2\Delta (0)\frac{(1+S(L))\Delta (L)+(1+S(0))\Delta (L)}{S(0)-S(L)}-\Bigg (1+\frac{2(1+S(L))}{S(L)-S(0)}\Bigg )\Delta (L)^2 \nonumber \\&\quad -\frac{1}{F}(S(L)-S(0))+\frac{\delta }{2}\Bigg (\frac{1}{(1-S(L))^2}-\frac{1}{(1-S(0))^2} \Bigg )+\sigma (S_{\xi \xi }(L)-S_{\xi \xi }(0))=0. \end{aligned}$$

For numerical purposes, it is simpler to specify the spatial period *L* and think of $\Delta (0)$ as the unknown quantity to be found as part of the nonlinear two-point boundary value problem. The present system allows for an exact evaluation of $\Delta (0)$ from (5.3) in contrast to iterations that would be typically required. For real admissible solutions, we require that $a_2^2-4a_1a_3>0$ where equation (5.3) has been represented as $a_1 \Delta (0)^2+a_2\Delta (0)+a_3=0$.

**Fig. 7:**
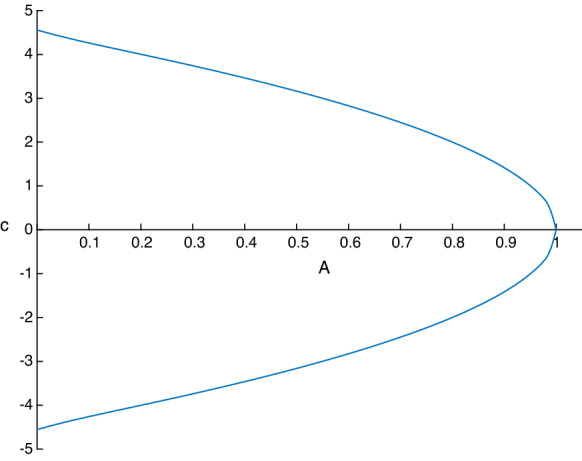
Relationship between amplitude and wave speed of periodic travelling waves with $L=0.5$, $S(L)=\Delta (L)=0$, $F=0.1$, $\delta =\sigma =0.01$

The conditions () give a relationship between the amplitude $A=|S(0)-S(L)|$ and the wave speed *c* of the periodic travelling wave solutions. In general, there are two branches of this relationship, giving two different speeds $c_1>c_2$ for a given amplitude. An example when the two waves have equal and opposite speeds is given in Fig. 7 for parameter values $F=0.1$, $\delta =\sigma =0.01$. The two branches in this case are symmetric due to the fact that the choice $\Delta (L)=0$ has been made, which implies from (5.3) that the (real) roots for $\Delta (0)$ have equal and opposite signs. It follows from expression (5.2a) that *c* has two equal and opposite values as seen in Fig. 7, where in addition we have fixed the length to $L=1/2$ and also took $S(L)=0$. Note that the choices $S(L)=\Delta (L)=0$ are natural for the recovery of solitary waves from periodic ones as we see later. The graph in Fig. 7 has been extended to the *y*-axis by using the linear dispersion relation:

5.4 $$\begin{aligned} c=\pm \sqrt{2 \sigma \left( \frac{\pi }{L} \right) ^2+\frac{2}{F}-2\delta }\, \end{aligned}$$

if $|S(0)-S(L)|\ll 0$, and to the *x*-axis by noting that as $c\rightarrow 0$ we have $S(0)\rightarrow -1$, i.e the wave touches the bottom channel wall for waves with zero speed.

While it is necessary to solve the full nonlinear system (5.1) numerically, it is useful to first obtain the linear solutions analytically and use them in numerical continuation to larger amplitudes. We look for linear travelling wave solutions in the form:

5.5a $$\begin{aligned} S(\xi )= & {} \frac{1}{2}\left[ S(0)+S(L)\right] +\left[ S(0)-S(L)\right] \,\widetilde{S}(\xi ), \end{aligned}$$

5.5b $$\begin{aligned} \Delta (\xi )= & {} \frac{1}{2}\left[ \Delta (0)+\Delta (L)\right] +\left[ S(0)-S(L)\right] \,\widetilde{\Delta }(\xi ), \end{aligned}$$

where the amplitude $|S(0)-S(L)|\ll 1$. Linearising the equations and looking for solutions $\widetilde{S}, \widetilde{\Delta }$ proportional to $\cos (\pi \xi /L)$ yields (note that having fixed the end conditions, the period 2*L* is an eigenvalue to be determined):

5.6a $$\begin{aligned} S&=\frac{1}{2}(S(0)-S(L)) \mathrm {cos}\left( \pi \xi /L \right) +\frac{1}{2}\Big (S(0)+S(L)\Big ), \end{aligned}$$

5.6b $$\begin{aligned} \Delta&=-\frac{1}{2}\frac{c+\Delta (0)+\Delta (L)}{1+S(0)+S(L)} \Big (S(0)-S(L) \Big )\mathrm {cos}\left( \pi \xi /L \right) +\frac{1}{2}\Big (\Delta (0)+\Delta (L)\Big ), \end{aligned}$$

where the frequency is given by

5.7 $$\begin{aligned} \left( \frac{\pi }{L}\right) ^2 = \frac{1}{\sigma }\left( \Big (c+\Delta (0)+\Delta (L)\Big )\frac{c+\Delta (0)+\Delta (L)}{1+S(0)+S(L)}-\frac{1}{F}+\frac{\delta }{\Big (1-\frac{1}{2}(S(0)+S(L))\Big )^3} \right) . \end{aligned}$$

**Fig. 8:**
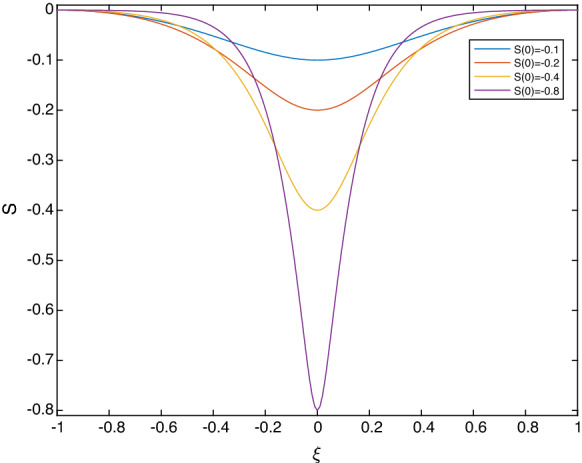
Periodic travelling waves as their amplitude |*S*(0)| increases (here $S(L)=\Delta (L)=0$). Blue curve – $S(0)=-0.1$; red – $S(0)=-0.2$; orange – $S(0)=-0.4$; purple – $S(0)=-0.8$. Other parameters are $F=0.01$, $\delta =1$, $\sigma =1$. (Color figure online)

**Fig. 9:**
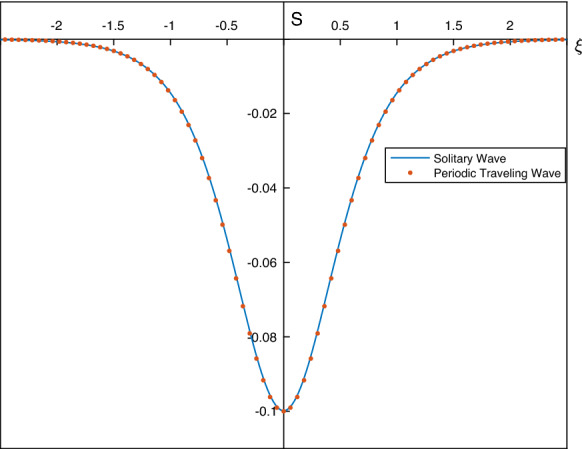
Comparison of a depression solitary wave of amplitude 0.1 calculated as in Sect. 4, with the corresponding periodic travelling wave in the long wavelength limit. Here, $S(L)=\Delta (L)=0$, and the other parameters are $F=\delta =\sigma =0.1$

In the results that follow, we fix $S(L)=\Delta (L)=0$ as discussed earlier and then construct periodic waves numerically for given *L* and amplitude *S*(0). Typical results for parameter values $F=0.01$, $\delta =1$ and $\sigma =1$, with fixed period equal to 2 (i.e. $L=1$) are given in Fig. 8. The waves depicted are those that tend to depression solitons and are generated by selecting $S(0)=-\,0.1, -\,0.2, -\,0.4, -\,0.8$. The smallest amplitude waves are in agreement with the linear results described above, but as the amplitude increases, we observe convergence to solitary waves. Such convergence is corroborated further in Fig. 9 for a slightly different set of parameters $F=\delta =\sigma =0.1$ that superimposes the depression solitary wave found using the analysis of Sect. 4. Agreement is seen to be excellent.

## Stability of periodic and solitary travelling waves

In this section, we consider a linear stability analysis of both the solitary wave solutions derived in Sect. 4 as well as the periodic travelling waves of Sect. 5. We shall perform this analysis in the case of a hydrodynamically passive upper fluid (i.e. $\alpha =0$) and the lower fluid a perfect conductor (i.e $\epsilon =0$) – the analysis can easily be extended to arbitrary parameters. The non-uniform travelling wave solutions in this case are governed by the coupled equations () with $\alpha =1, \epsilon =0$, and we denote them by $\overline{S}(\xi )$ and $\overline{\Delta }(\xi )$, recalling that they travel with speed *c* and $\xi =X-cT$. We perturb these solutions so that

6.1 $$\begin{aligned} S(\xi ,T)=\overline{S}(\xi )+S'(\xi , T), \quad \Delta (\xi ,T)=\overline{\Delta }(\xi )+\Delta ' (\xi , T), \end{aligned}$$

where $|S^\prime (\xi ,T)|, |\Delta ^\prime (\xi ,T)|\ll 1$, substitute into the governing equations (3.15) and linearise to find

6.2a $$\begin{aligned}&S_T'-cS_\xi '-2(\overline{\Delta } S'+ \Delta ' \overline{S})_\xi -2\Delta _\xi '=0, \end{aligned}$$

6.2b $$\begin{aligned}&\Delta _T' -c \Delta '_\xi -2(\overline{\Delta } \Delta ' )_\xi -\frac{1}{F} S'_\xi +\frac{ \delta }{(1-\overline{S})^3}\left( S'_\xi +\frac{3\overline{S}_\xi S'}{1-\overline{S}}\right) =-\sigma S_{\xi \xi \xi }'. \end{aligned}$$

We assume that the perturbed solutions are of the form

6.3 $$\begin{aligned} S'=\mathrm{{e}}^{\lambda T}\mathrm{{e}}^{ik \xi }\widetilde{S}(\xi ), \quad \Delta '=\mathrm{{e}}^{\lambda T} \mathrm{{e}}^{i k \xi }\widetilde{\Delta }(\xi ), \end{aligned}$$

where $\widetilde{S}(\xi )$ and $\widetilde{\Delta }(\xi )$ are 2*L* periodic and the wavenumber $0\le k\le \pi /L$ allows perturbations that are longer than 2*L*, e.g. subharmonic. In what follows we take $k=0$, i.e. we consider waves with the same wavelength as the basic period, and use these results with comparisons with corresponding solitary wave stability later. Substituting (6.3) into (6.2a)–(6.2b) (with $k=0$), yields the following eigenvalue problem to determine $\lambda$:

6.4a $$\begin{aligned}&c\, \widetilde{S}_\xi +2(\overline{\Delta }_\xi \, \widetilde{S}+\overline{\Delta }\, \widetilde{S}_\xi +\overline{S}\,\widetilde{\Delta }_\xi +\overline{S}_\xi \,\widetilde{\Delta }+ \widetilde{\Delta }_\xi )=\lambda \widetilde{S}, \end{aligned}$$

6.4b $$\begin{aligned}&c\,\widetilde{\Delta }_\xi -\sigma \, \widetilde{S}_{\xi \xi \xi }+2(\overline{\Delta }\, \widetilde{\Delta }_\xi +\overline{\Delta }_\xi \,\widetilde{\Delta })+\frac{1}{F}\widetilde{S}_\xi -\frac{\delta }{(1-\overline{S})^3} \left( \widetilde{S}_\xi +\frac{3\overline{S}_\xi }{1-\overline{S}}\,\widetilde{S} \right) =\lambda \widetilde{\Delta }. \end{aligned}$$

The eigenvalue problem () is solved numerically after specifying periodic boundary conditions. Finite difference methods are used, and the system is cast into a matrix eigenvalue problem of the form $A\mathbf {r}=\lambda \mathbf {r}$ where $\mathbf {r}$ is the discretisation of $\widetilde{S}, \widetilde{\Delta }$ on the grid.

**Fig. 10:**
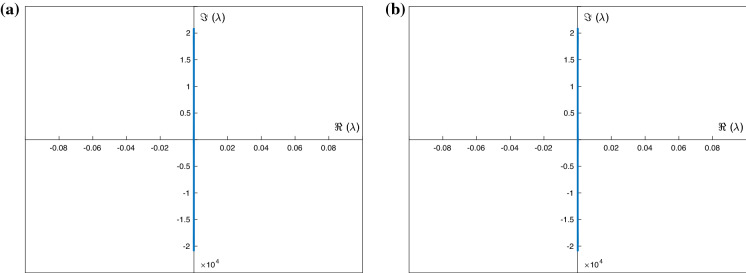
Stability spectra of the travelling waves in Fig. 9: **a** periodic travelling wave, **b** solitary wave

We begin with the stability of the depression waves shown in Fig. 9, recalling that we have superimposed a calculated solitary wave along with its large wavelength analogue. We have carried out the stability by using null conditions far away for the solitary wave profiles, as well as spatially periodic boundary conditions for large enough periods. The resulting spectra are the same and shown in Fig. 10. It can be seen that all eigenvalues lie on the imaginary axis, and hence, the waves are neutrally stable. The vertical extent of the spectrum increases as resolution increases and higher wave numbers enter into the calculation—this is expected due to the dispersive regularisation provided by surface tension. We also note that according to the criterion (4.30), the waves in Fig. 10 are what we termed hyperbolic, i.e. the eigenvalues of the accompanying nonlinear flux matrix are real, and this provides an explanation for the stability found in our computations. In fact, all wholly hyperbolic depression solitary waves studied were found to be neutrally stable, and hence, criterion (4.30) can be used as a simple rule of thumb to determine linear stability (albeit to waves with wavelengths at most 2*L*—modulational stability will be considered elsewhere). The converse of this rule of thumb also holds: namely, if the wave is locally elliptic, then the spectrum contains at least one eigenvalue with positive real part and the system is unstable. As all elevation solitary waves are proven to be at least locally elliptic, these are found to be unstable.

**Fig. 11:**
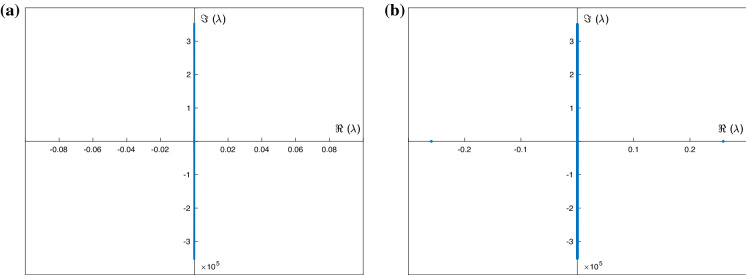
Stability spectra of the periodic travelling waves in Fig. 8: **a** Smaller amplitude $S(0)=-0.1$, **b** larger amplitude $S(0)=-\,0.8$

**Fig. 12:**
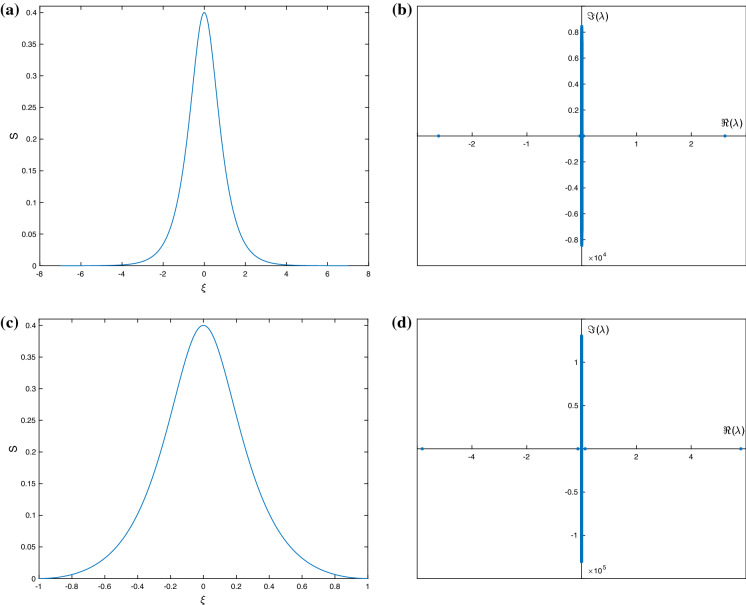
Examples of elevation periodic travelling waves and their stability spectra. Top row: $F=0.1, \ \delta =5, \ \sigma =1$; **a** the profile, **b** its spectrum. Bottom row: $F=0.1, \ \delta =5, \ \sigma =0.1$; **c** the profile, **d** its spectrum. The waves are unstable – there is an eigenvalue $\lambda >0$ in both cases

**Fig. 13:**
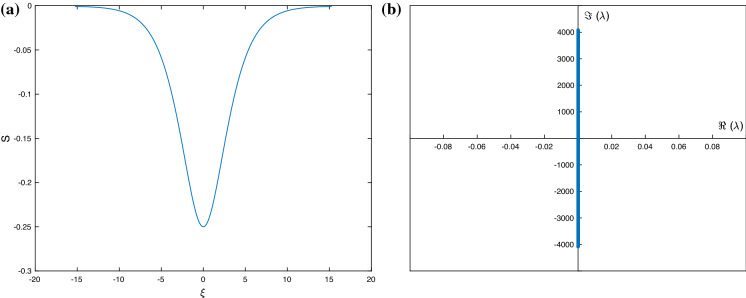
Explicit solitary wave in the absence of an electric field having amplitude $-1/4$—see formulas (54)–(55) in [16]. **a** The depression solitary wave. **b** Its spectrum showing neutral stability. Parameter values are $F=c=\sigma =1$

The effect of increasing the amplitude of periodic depression waves of permanent form was investigated in Fig. 8 for $F=0.01$, $\delta =1$ and $\sigma =1$. For the stability of those waves, in Fig. 11 we give results for the smallest amplitude wave having $S_{min}=-0.1$, and largest amplitude one having $S_{min}=-0.8$. The results show that the smaller wave is neutrally stable, whereas the larger one has one unstable mode with real part $\approx 0.259$. We note that the number of unstable modes increases as the surface tension $\sigma$ is decreased (not shown) due to the reduced dispersive regularisation when surface tension is weaker.

Turning to elevation waves next, in general, we find that these are typically more unstable than depression waves for a given set of parameters and amplitude. The instability behaviour is analogous to that of depression waves, in the sense that longer waves appear to be more stable that shorter ones having the same amplitude, and larger amplitude waves are also more unstable. Typical results are included in Fig. 12 for parameters chosen to give a long waves of period 14 units (essentially solitary), and a shorter wave of period 2 units, both of relatively large amplitude equal to 0.4. Panels (a) and (b) show the solitary wave and its spectrum, with panels (c) and (d) depicting the finite-period wave and its instability characteristics. Both of these waves are wholly elliptic according to the criterion (4.30), and hence, it is not surprising to observe unstable eigenvalues. We note that as the surface tension parameter $\sigma$ is increased, the waves become more sinusoidal and also linearly stable (this was confirmed but not shown for brevity).

Finally, we consider the stability of the non-electrified solitary waves that were reported in [16]. These were constructed for different parameters but their stability was not investigated. Our computations indicate that all the solitary waves in [16] are neutrally stable, and we illustrate this for the case $\alpha =1$ (upper layer is hydrodynamically passive). To switch off the field in our model, it is sufficient to take $\epsilon =1$ and $\delta =0$, construct solitary waves as in Sect. 4.2.1, and study their stability according to the eigenvalue problem (). As noted in [16], depression solitary waves alone exist, and calculation of the eigenvalues of the flux matrix gives real values only, hence, the waves are what we termed of hyperbolic type. Their stability is analogous to the $\epsilon =0$ case considered in Sect. 4.2.2. The profile for one of the exact solutions found in [16] and its spectrum, showing neutral stability, are given in Fig. 13 for the set of parameters $F=c=\sigma =1$. The exact explicit solution used here is given by Eqs. (54)–(55) in [16].

## Conclusions

In this paper, we considered a two-fluid flow in a horizontal channel and allowing for the effects of gravity, electrical fields, and surface tension. We derived a $2\times 2$ system of nonlinear 1-D evolution equations by carrying out a fully nonlinear long-wave asymptotic analysis that describes wave amplitudes scaling with the channel thickness. The conditions under which solitary wave solutions exist were derived in the case of a lighter upper fluid (i.e. a Rayleigh–Taylor stable regime), and the role of the electric field was elucidated. In the case when the upper fluid has negligible density (i.e. is hydrodynamically passive), and the lower fluid is a perfect conductor, it was shown that two solitary waves are always produced, an elevation and a depression wave, having the same speeds. Using the eigenvalues of the nonlinear flux matrix of the system, all travelling waves were classified to be locally elliptic or hyperbolic depending on whether the local eigenvalues are complex or real, respectively. The zero surface tension limit recovers the equations of [5], and the elliptic/hyperbolic diagnosis was extended to more general dielectric fluids where $\epsilon _-\ne \infty$. Periodic travelling waves were also constructed and the relationship between amplitude and wave speed was investigated, prior to carrying out a linear stability analysis of both classes of non-uniform solutions. In general, depression solitary and periodic waves were found to be stable whereas elevation ones can be unstable if the surface tension parameter is not too large. Our results show, as expected that the presence of locally elliptic regions in the travelling waves produces instability, and a necessary condition for neutral stability is that the profiles are locally hyperbolic over the whole spatial period. Future directions include the investigation of disturbances that are longer than the basic period of travelling waves (modulational instabilities), and a more complete study of shock type solutions involving heteroclinic connections and their stability.
